# Development and implementation of an on-demand competency-based onboarding program for clinical research professionals in academic medicine

**DOI:** 10.3389/fmed.2023.1249527

**Published:** 2023-12-22

**Authors:** Jessica R. Cranfill, Christine E. Deeter, Deborah Hannah, Denise C. Snyder, Stephanie A. Freel

**Affiliations:** ^1^Duke Office of Clinical Research, Duke University, Durham, NC, United States; ^2^School of Medicine, Duke University, Durham, NC, United States; ^3^Clinical and Translational Science Institute, Duke University, Durham, NC, United States

**Keywords:** clinical research professional, clinical research coordinator (CRC), competency-based training, onboarding program, E-learning, workforce development, professional development, instructional design

## Abstract

Over the past 7 years, Duke has implemented competency-based job classifications for clinical research professionals (CRPs) with a defined pathway for career advancement. The workforce is defined specifically as the collection of staff employed across the clinical research enterprise to operationalize clinical research and human participatory protocols through the hands-on conduct of protocol activities including participant enrollment, regulatory coordination, study documentation, data collection and management, and sponsor engagement. The competency framework for this critical workforce laid the foundation for a centrally developed on-demand onboarding program at Duke. The self-paced program is designed to engage learners through competency-based learning modules, guided mentor/manager discussions, and applied learning activities. Consisting of an initial E-Learning orientation to clinical research at Duke, called Express Start, followed by a 90-day role-based Onboarding Learning Plan, our onboarding program includes training in foundational pre-defined core competency areas and customizable learning paths. Associated Engagement Activity Packets for many clinical research competencies encourage mentor and/or manager involvement and hands-on learning for the employee through suggested enrichment activities. The program has been widely adopted for CRPs within the Duke University Schools of Medicine and Nursing, and newly hired CRPs and their managers have expressed satisfaction with these centrally offered tools. In this paper, we describe the methods used to develop and implement our competency-based onboarding program. We will share an evaluation of the program and planned next steps for expanding the suite of onboarding resources.

## 1 Introduction

Coordination and management of clinical research projects and programs within academic medicine has become increasingly recognized as a profession over the past decade (1, 2). As study conduct has become more complex, a growing number of tasks (informed consent, regulatory submissions, addressing privacy and data, engaging diverse community populations for recruitment, etc.) are delegated to these clinical research professionals (CRPs) (3). Yet, academic medical centers (AMCs) often struggle to identify, train, and develop this critical workforce within their institutions due to several factors including limited resources for staff-level managers and poorly defined clinical research workforce structure (4, 5). The consequent nebulous workforce is difficult to identify and oversee, ultimately impacting site quality. A multi-institutional task force convened to address the burgeoning complexity and lack of job standardization across CRP workforces resulting in the Joint Taskforce for Clinical Trial Competency framework, published in 2014 (6). Further efforts under Duke’s Workforce Engagement & Resilience Initiatives (WE-R) to standardize clinical research jobs and career ladders at Duke with distinct JTFCTC competency-based roles have reduced attrition and created a CRP identity across twelve jobs (1, 2, 5–11). Consequently, the WE-R initiatives housed within the Duke Office of Clinical Research (DOCR) laid the necessary groundwork for developing competency-based onboarding for newly hired CRPs. The WE-R team began aligning CRP training opportunities with the JTFCTC framework (6) in 2018 and incorporating competency learning into the onboarding process to strategically propel CRPs toward competency-based thinking. This enables them to consistently progress within the established leveled framework, allowing for the enhancement of their competencies through continuing educational opportunities and positioning them to take advantage of advancement opportunities (7, 8, 11). We believe that onboarding programs with a foundation in this widely recognized competency framework will fill a critical gap in CRP development at many AMCs.

Through our work with the Association for Clinical and Translational Science (ACTS) Clinical Research Professional Taskforce (CRPT) Special Interest Group, including professional partners from the Association of Clinical Research Professionals (ACRP) and Clinical and Translational Science Award (CTSA) institutional networks, it is evident that effective onboarding and training paradigms remain a significant need in CRP workforce development (5). Onboarding in this context is the development of fundamental competencies that allow CRPs to perform their job successfully. The onboarding period for a new hire often aligns with a 3 to 6-month evaluation period and may be a constant endeavor for some teams due to a combination of portfolio growth and staff turnover. This can be a costly process; estimates for the cost of turnover for one employee can top $50 K or more, not including lost revenue related to study pauses, and can lead to manager burnout (8, 11). Importantly, poor onboarding may contribute to an unsupportive research culture and leave new staff without the ability to demonstrate necessary competency skills leading to more turnover early in the new hire period. We initiated our WE-R Onboarding Program to standardize CRP onboarding using established competencies and to ameliorate the onboarding burden faced by study teams and managers of new CRPs.

When job classifications are not standardized, it can be difficult to estimate how many CRPs are hired each year nationally or globally (12). Our twelve standardized job classifications combined host between 800–900 staff at a time and include entry-level Clinical Research Specialists, Clinical Research Coordinators, Regulatory Coordinators, Clinical Research Nurse Coordinators, Research Program Leaders, and senior-level and CRU management positions. Such standardization has allowed us to track attrition and hiring metrics over time, showing that in 6 years between FY2017 through FY2022, we have hired more than 1,200 new CRPs into Duke (11). Duke has over 24 clinical research units (CRUs) that are largely defined by clinical therapeutic area (e.g., Population Health, Pediatrics, Oncology, etc.) and vary in size of workforce based on research portfolio. Across all of our CRUs, we identified two essential problem areas relating to the onboarding of new CRP staff: (1) lack of standard and up-to-date onboarding tools for specific CRP positions, and (2) lack of alignment with established CRP competency domains. A series of “Un-Meetings,” hosted by the ACTS and led by the CRPT Special Interest Group has uncovered the pervasiveness of these two critical deficits across both AMCs and Contract Research Organizations (CROs) (5). At Duke, we found that the onboarding tools used within the CRUs were inconsistent, quickly became outdated, and lacked a clear connection to an established professional competency framework. This lack of competency alignment created the potential for performance inequities across defined roles and unmet expectations for employees working across CRUs and therapeutic areas. Moreover, fully decentralized onboarding multiplied the effort required for managers to maintain onboarding processes and materials. The existing tools, developed and maintained by individual managers or units, did not align with the JTFCTC competency framework, resulting in knowledge gaps that might only be discovered later when CRPs needed to apply their skills. By centralizing and standardizing CRP onboarding tools at Duke, we provided managers with a structured onboarding plan that covered all the essential competency areas for their new employees’ roles, with training materials that were kept up to date. This method allowed us to leverage the expertise of a dedicated instructional designer who specializes in adult learning to create an effective learning framework. Furthermore, by defining job-based core competencies that transcend research area, as well as distinct competency paths that align with more research area-specific requirements, we have created a foundational learning platform that can be tailored to, and grow with, the employee’s career. Although our tools were developed in the context of Duke’s CRP career structure, the transcendency of work roles, onboarding challenges, and the JTFCTC competency framework allowed us to create an onboarding program that can be easily adapted and widely implemented across clinical research sites and AMCs.

## 2 Pedagogical framework

We intentionally crafted our onboarding program to be flexible for CRPs and their managers and incorporated key elements for self-paced adult learning. These elements included a digital learning strategy with online modules to present new information and guided applied learning tasks. We reviewed onboarding models across the field, seeking a balanced approach that promotes competency-based learning retention while remaining flexible and feasible for our small, centralized team to manage. During our assessment of onboarding models, ranging from boot camps to fully centralized, training-intensive programs, we observed that boot camps—intensive training over a short period—are commonly provided (13). While this option provides significant and often competency-aligned training, this accelerated method may not be as conducive to information retention as more gradual, on-the-job onboarding options, due to the absence of experiential learning and hands-on practice (14).

To evaluate options that might better promote competency-based learning retention, we engaged collaborators across CTSA CRPT Special Interest Groups to understand onboarding programs being offered among our peer institutions. The challenges of implementing a fully centralized program, such as that written by Musshafen et al. (15), include both the significant training effort and expense needed to run the program (multiple dedicated full-time staff for 200+ new hires each year) and the amount of time new employees spend away from their projects during the onboarding period. We aimed to create and broadly share a hybrid approach that capitalizes on the most effective strategies from each of these successful models. While not a factor when we began program development, the need to accommodate onboarding during the remote and hybrid environments of the COVID-19 pandemic quickly became an important influence in program design and remains advantageous for the growing number of decentralized research teams. To our knowledge, there are no published or disseminated tools from other institutions for a similar on-demand and competency-based onboarding program for the CRP workforce.

**Figure 1:**
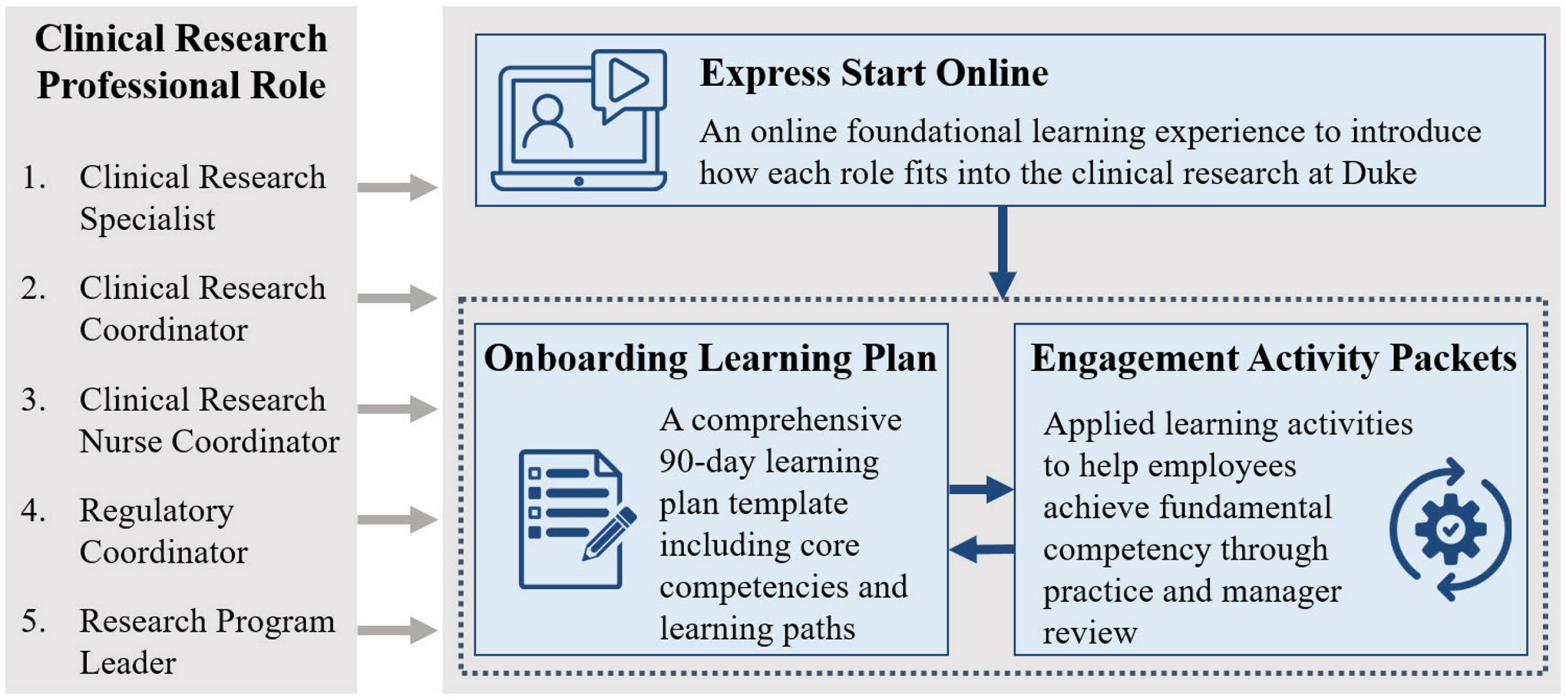
The clinical research professional onboarding program at Duke consists of 3 parts: (1) Express Start is an online foundational learning experience to introduce how each role fits into clinical research at Duke, (2) the Onboarding Learning Plan is a comprehensive 90-day learning plan template including core competencies and learning paths, and (3) the Engagement Activity Packets include applied learning activities to help managers guide the onboarding process and help employees achieve fundamental competency through practice and manager review.

Program development relied on partnerships between the central WE-R team, CRU leaders, and individual managers. To ensure the program was both impactful and acceptable across CRUs, we explored needs with CRU leaders and considered the versatility of tasks and work locations. Using our existing competency framework, we developed a semi-centralized onboarding program for each of our CRP job classifications which includes several components (see Figure 1). Due to the decentralized and federated CRU structure at Duke, direct supervisors and managers are responsible for the supervision and mentorship of newly hired CRPs, implementation of the onboarding program and tools into their CRU’s onboarding practices, and management of competency acquisition and advancement for their CRPs. While the use of this program is currently not required across the institution, it is a step toward standardizing training for clinical research staff and preparing them for recurrent competency development and career advancement. The following sections describe our work to centrally develop, implement, and evaluate this onboarding program for CRPs at Duke University.

## 3 Learning environment

The competency-based CRP onboarding program at Duke is driven by employees and their managers and includes several components that work together to prepare CRPs for their careers at Duke. We used an iterative process to develop each component and sought input from members of the research community throughout the process.

### 3.1 Mapping existing training to competencies

Before developing centralized onboarding, our first step was to map existing relevant training into the competency framework at Duke. This allowed us to understand existing resources, identify training gaps, and begin to frame a competency-based training structure. A total of 97 existing courses were mapped to clinical research competencies. Mapped trainings were developed by the Duke Office of Clinical Research (DOCR), Duke Clinical and Translational Science Institute, Duke Office of Physician Scientist Development, Duke Office of Scientific Integrity, Duke Occupational and Environmental Safety Office, Duke Office of Regulatory Affairs and Quality, the Collaborative Institutional Training Initiative, and the Society of Clinical Research Sites (16, 17). The process of mapping training to clinical research competencies was useful to (1) identify currently available training in each competency area, (2) organize courses appropriately for staff to easily identify training needed for skill growth, (3) pinpoint gaps and prioritize opportunities for new course development, and (4) inform competency-based onboarding development.

### 3.2 Engaging the Duke clinical research community and leadership

Buy-in and engagement from the clinical research community and executive leadership were essential in developing an onboarding process applicable across all Duke CRUs. To achieve this, we assembled a steering committee including our DOCR WE-R team, Duke School of Medicine Human Resources, and colleagues from CRUs of various sizes and therapeutic areas. The committee’s goal was to contribute to an onboarding program that represented Duke as an entity yet easily tailored to meet specific CRU needs.

To capture specific needs across every CRU, we surveyed CRU leadership via REDCap (an electronic data capture tool described in detail in the Acknowledgments section of this paper) (18). The survey queried which of the JTFCTC competencies CRU leaders considered necessary for a given job role within their unit, what they desired in a centrally developed onboarding program, and how they onboarded new CRPs at the time. Consistent themes included the need for onboarding tools that: (1) introduced the competency framework and promoted competency development, (2) were centrally maintained and regularly updated, (3) were standardized for each CRP job role, yet flexible and customizable for unit-specific functions, (4) could be available on-demand for new staff to begin immediately upon start, (5) included a manageable and adjustable timeline for completion, and (6) encouraged application of concepts on the job with intentional manager involvement.

### 3.3 Addressing the heterogeneous academic research environment

As expected, survey results showed variability regarding which competencies were considered essential across CRUs. However, follow-up interviews with CRU leaders to further discuss the survey identified several common competencies for each job role despite differences in research areas and project types. One essential goal was, therefore, to develop a tool that was both standardized for these core job competencies and flexible for our varied research areas. To address this, we created an onboarding framework with required “core competencies” and elective “learning paths” for each CRP role.

- **Core competencies:** competencies deemed necessary for all individuals in the job classification across all research areas.
- **Learning paths:** chosen by the hiring manager based on an individual CRP’s job responsibilities and research environment. Individuals are likely to have multiple paths based on the job functions they need to learn.

To ensure universally applicable categorization, the WE-R team interviewed each survey respondent individually to discuss the initial survey results and achieve consensus on the defined cores and learning paths. During these interviews, the team also gathered the current onboarding plans and checklists used across units to further compare consistency and variations in onboarding across the units. Finally, core competencies and learning paths were presented at CRU leadership meetings to obtain consensus for the Clinical Research Coordinator (CRC) and Regulatory Coordinator (RC) roles. We attribute much of the voluntary uptake of standardized onboarding to the engagement of CRU leadership and CRPs throughout the development of the program.

At the end of fiscal year 2022, the CRC job classification accounted for 40.89% of the Duke CRP workforce. The greatest number of annual hires are also in CRC jobs. Clinical Research Specialist Srs. and Clinical Research Specialists (CRS) combined accounted for 17.18% of the CRP workforce. Therefore, we began by developing onboarding tools for CRCs and then modifying them for CRSs. Table 1 is a visual representation of the core competencies and learning paths defined for each role’s onboarding.

**Table 1:** This table shows the competencies included in the Onboarding Learning Plan (OLP) template for each clinical research professional job described in this paper.

Job title	Core competencies: foundational learning applicable to everyone in the role	Learning paths: competencies chosen by the manager based on responsibilities
Clinical Research Specialist (CRS)	Express Start for CRS Electronic Management of Participants* Participant Level Documentation* Data Security and Provenance* Institutional Regulatory Policies and Procedures*	Recruitment* Databases Adverse Events* Contracts and Agreements Study Closeout*
Clinical Research Specialist, Senior (CRS Sr.)	Express Start for CRS Sr. Electronic Management of Participants* Participant Level Documentation* Data Security and Provenance* Institutional Regulatory Policies and Procedures*	Consent Procedures* Recruitment* Databases Adverse Events* Contracts and Agreements Study Closeout*
Clinical Research Coordinator (CRC)	Express Start for CRC Electronic Management of Participants/Protocols* Consent Procedures* Participant & Study Level Documentation* Data Security and Provenance* Institutional Regulatory Policies and Procedures*	Recruitment and Screening* Databases Adverse Events* Regulatory Cores* Investigational Products* Specimen Handling Study Closeout* Financial-Related Training
Clinical Research Nurse Coordinator (CRNC)	Express Start for CRNC Duke Health Nursing Orientation & Nursing Competency Checkoffs Electronic Management of Participants/Protocols* Consent Procedures* Participant & Study Level Documentation* Data Security and Provenance* Institutional Regulatory Policies and Procedures*	Recruitment and Screening* Databases Adverse Events* Regulatory Cores* Investigational Products* Specimen Handling Study Closeout*
Regulatory Coordinator (RC)	Express Start for RC Electronic Management of Protocols* Development of Informed Consent Documentation and Plan* Navigating the Ethics Review Process Institutional Regulatory Policies and Procedures* Data Security and Provenance* Sponsor/Regulatory Reporting	Participant and Study Level Documentation* Databases Adverse Events* Contracts and Agreements* Study Closeout* FDA Regulatory Submissions*
Research Program Leader (RPL)	Express Start for RPL Electronic Management of Participants and Protocols Operational Leadership (Institutional Regulatory Policies and Procedures, Leading Project/Program Staff, Budgeting and Resource Management*) Project Management (Project Initiation and Scope*, Project Planning*, Stakeholder Management*, Task Management*, Milestone Tracking and Reporting) Intellectual Contribution and Scientific Concepts* *(Proposals, Grants, Manuscripts, and representing the program)* Leadership and Professionalism *(Professional Development, External Awareness, Organizational Agility, Resilience and Adaptability, Subject Matter Expertise and Problem Solving, Communication and Teamwork)*	Contracts and Agreements Investigational Products* Study Documentation* Recruitment* Participant Retention Monitoring and Audits Adverse Events* Informed Consent* Navigating the Ethics Review Process Sponsor/Regulatory Reporting Data Security and Provenance* Data Collection and Entry Coordination with Sponsor/CRO Study Closeout*

The OLP template for each job title includes core competencies that are relevant to anyone new to the role as well as learning paths that are chosen by the manager based on individual responsibilities. Competencies with an asterisk (*) have an associated Engagement Activity Packet.

### 3.4 Onboarding program components

To meet the described needs of our CRUs, including the hybrid work environment required during the COVID-19 pandemic, the WE-R team started developing three primary onboarding components that are available on demand. The components include (1) Express Start Online, (2) Onboarding Learning Plan, and (3) Engagement Activity Packets.

#### 3.4.1 Express start

Express Start is a series of self-paced E-Learning modules for each role that serves as an introduction to clinical research and the competency framework at Duke. This introduction is meant to provide context for all additional training tasks a new employee will complete, allowing them to relate what they learn to their understanding of their role within the institution. These modules include an overview of the clinical research competencies, activities, regulations, and workflows specific to their role, and provide a sense of what other members of their team may be responsible for. Refer to Supplementary Table S1 for a list of the modules included in Express Start for each role.

#### 3.4.2 Onboarding learning plan templates

The Onboarding Learning Plan (OLP) template for each role provides a curated list of technical online (and limited in-person) training to develop fundamental skills in the CRP competencies. Core competencies and customizable learning paths are organized within the easily personalized template, refer to Table 1 for cores and paths mapped to each CRP role. Adult learning motivation stems from understanding how learning will be applied and valuing learning outcomes (19). Therefore, we provide a 90-day week-by-week plan that can be aligned functionally and temporally with work activities. The OLP templates include core learning for everyone in the role, learning paths that may be relevant to the role, and space for the manager to add any additional study or unit-specific training requirements for the employee. The core competencies for each position set the stage for foundational job activities in clinical research that are necessary to perform a CRP job and apply across all therapeutic areas. The inclusion of customizable learning paths in the Onboarding Learning Plan recognizes the diverse responsibilities CRPs may have in different therapeutic areas and allows for tailored learning experiences while maintaining standardization to the JTFCTC competency area. This flexibility ensures that the onboarding program caters to the specific needs of individual CRPs, acknowledging the varied competencies required across research areas.

The OLP templates have a customizable week-by-week timeline with a checklist structure so the employee can check off training items upon completion. Each week includes training for one to two clinical research competencies and an estimated time to complete certain tasks. Weekly goals provide the employee with a sense of structure, space out learning over time, and allow the employee allotted time to do hands-on activities related to each competency. Managers are encouraged to review the timeline and organize the plan in a way that aligns training with the employee’s opportunity to practice certain tasks.

The OLP includes a description for the CRP of each learning element listed. This description provides necessary context that helps orient them by clarifying the purpose for specific tasks and workflows. Courses that are required by institutional policy are marked as such. While this context can help ensure employees only complete courses that apply to their responsibilities, the manager is expected to review the template and tailor it to the needs of the specific employee before providing it to them.

#### 3.4.3 Engagement activity packets

Engagement Activity Packets are available for many of the clinical research competencies and are linked within the OLP. This tool is used by managers to guide supervision of the onboarding process and mentoring of new employees as they work to acquire each competency. The Engagement Activity Packets include the following elements to help the employee achieve fundamental competency through application, practice, and manager review:

1. **Knowledge objectives and fundamental skills:** A description of expected knowledge and skill after 90-day onboarding. These are tied to the established fundamental skill level for the competency (8).
2. **Recommended guidance, policies, and additional courses**: A list of recommended guidance (websites, resources, etc.), policies, and courses to supplement those included in the OLP as needed.
3. **Manager review questions:** Guided questions to review with a manager or mentor. These are intended to engage the manager in the learning process and keep them informed of progress and opportunities for clarification.
4. **To-do items and suggested shadowing activities:** Suggested activities to help the employee make the connection between E-Learning courses and their daily work. These provide a means for meaningful team interactions and experiential learning. *The involvement of a manager/mentor in the onboarding process is critical for this piece to be effective.*

The combination of the three onboarding components described above offers a foundational starting point for career competency development and structured reference materials for many job tasks. These tools can be used for new hires to Duke, transfers between units, and staff with limited experience in clinical research. The flexibility of the onboarding toolkit as a whole allows managers to personalize the onboarding process depending on an employee’s existing strengths and experience.

### 3.5 E-learning course development and rationale for on-demand training

It is important to note that many of the courses included in this onboarding program are asynchronous E-Learning modules. Most of the modules were developed by an instructional designer on the DOCR WE-R team using Storyline and Rise authoring software, which are both included in the Articulate 360 E-Learning development platform’s suite of tools used for instructional design (4). Subject matter experts from CRUs and DOCR were consulted in the design, development, and review of each module. Modules include an engaging combination of reading, narration, video, interactivity, practice, and assessment.

The shift to on-demand E-Learning modules rather than live, instructor-led courses occurred for several reasons. The volume of new hires in clinical research positions has led to an overwhelming demand for training (roughly 200 annually). At the same time, CRPs are located across hospitals, clinics, and in the community with limited time or ability to attend in-person training. E-Learning modules are a solution for training that does not require an in-person observation of competency. On-demand modules afford CRPs the ability to learn at their own pace, intersperse study-specific training as needed, and spend more time upfront practicing job-specific tasks with their study team. Additionally, the ability to access content within these modules at any time has allowed for more just-in-time training and guidance for the whole clinical research community.

The creation of E-Learning modules is a time-intensive endeavor up front, but centrally maintaining, hosting, and tracking training completion is more simplified and less effort-intensive in the long term. The limitation to exclusively using E-Learning in the complex clinical research environment is the need for hands-on practice to retain skills in many competencies (20). As described above, the Engagement Activity Packet component of our onboarding program addresses this pitfall by providing a means for applying the competencies on the job.

## 4 Implementation of the onboarding program

Institutions that are interested in accessing the onboarding program tools described above may request access to our repository of licensed materials and an Onboarding Toolkit Implementation Plan. There is a request form publicly available on the Duke Office of Clinical Research Onboarding and Training for Clinical Research Professionals website.^1^ The implementation plan further details the program components, audience, competency-based jobs foundation, program intention and goals, program team, software used, description of program components and materials, program implementation steps, challenges and solutions, manager guidance, and a full implementation timeline.

### 4.1 Phased launch

**Figure 2:**
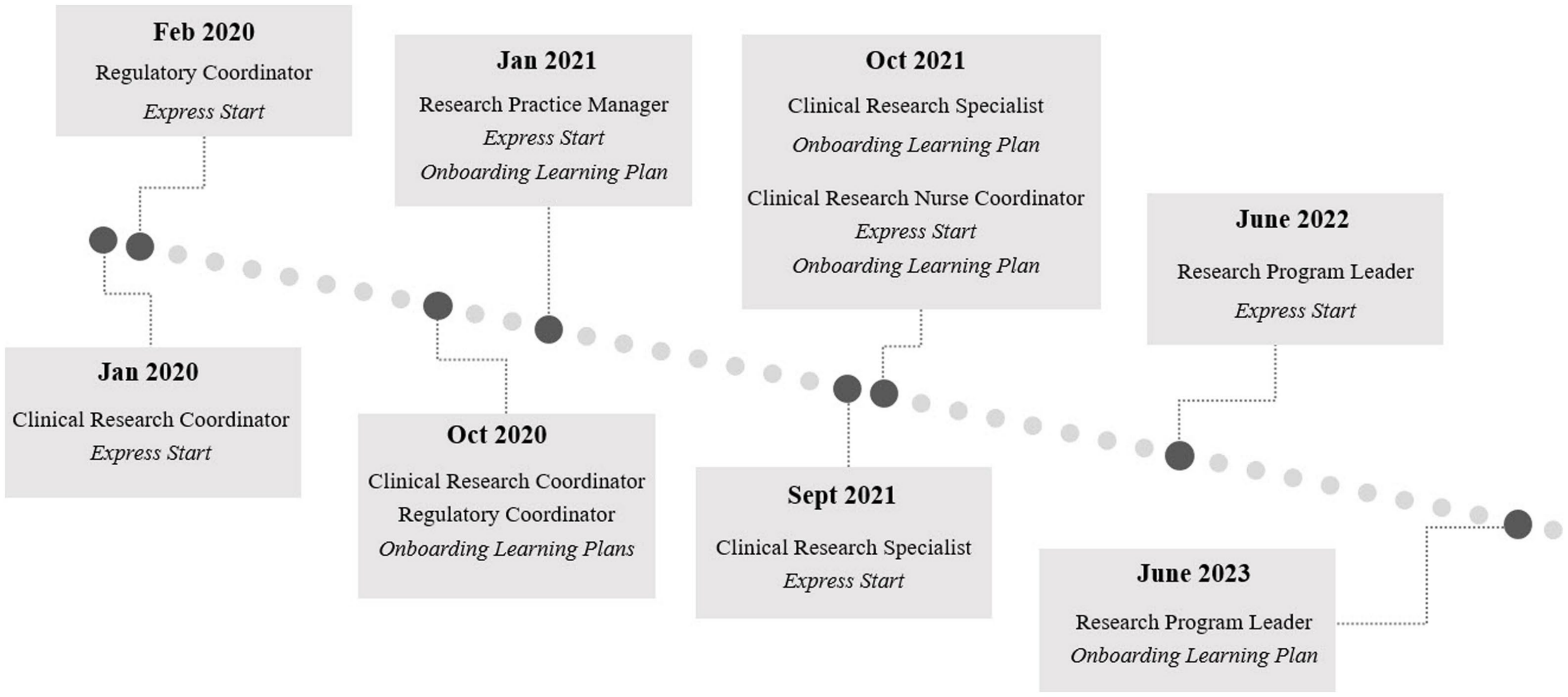
Onboarding program implementation and launch timeline for each component of the Duke onboarding program for clinical research professionals by role. Engagement Activity Packets launched in October 2020 and June 2023 along with the Clinical Research Coordinator and Research Program Leader Onboarding Learning Plans, respectively. The phased development and launch of the tools spanned from 2020 to 2023 and iteration will continue.

Implementation of the full suite of onboarding tools for all CRP jobs at Duke occurred in phases, with tools for each role launching as development concluded. The full timeline for the launch of each element from 2020 to 2023 is illustrated in Figure 2.

We initially planned a soft launch of the full suite of onboarding tools in mid-2020 with 43 staff in CRC and Regulatory Coordinator roles across nine volunteer CRUs. Those in this phase would receive a survey at 30, 60, and 90 days about the program and their comfort with the competencies covered. However, several weeks into the soft launch, as more CRPs were hired, we began receiving requests from additional managers who were not participating in the first phase of the rollout to receive the tools. As teams were managing COVID-19 demands during this time, the increasing need for an online, standard solution for managers to onboard and train staff was apparent. We discontinued the pilot evaluation phase to focus on disseminating and training all interested managers to use the tools. We later reconvened to assess the program as described in the Program Assessment.

### 4.2 Implementing the program components

The primary mechanism for delivery of the program components is the Duke Office of Clinical Research WE-R website where managers can locate all information associated with using these tools. Each tool is either housed in Duke Box^2^ (secure cloud-based storage and collaboration service), the Duke Learning Management System (online system for training management and completion tracking), or can be requested directly from the WE-R team. The website and materials are maintained by the DOCR Manager of Education and Outreach/Instructional Designer.

When a new hire is identified, their manager downloads the most recent version of the plan from the WE-R website and adjusts the template as needed to align with opportunities for hands-on application and onboarding timeline needs. This includes choosing relevant learning paths for the employee, indicating a goal week for completion of each competency, and removing any learning paths that are irrelevant to the employee’s research focus. For onboarding to be most effective, we recommend managers meet regularly with the new employee throughout the 90-day onboarding period to review the Engagement Activity Packets, keep up with progress, and adjust timelines or learning paths as needed.

### 4.3 Communication and support

Availability of the new onboarding tools for CRPs was initially communicated to CRU leadership who relayed information about the program and provided their expectations for use to managers in their unit. Announcements to the full community occurred via our Clinical Research Update Newsletter, targeted email announcements from clinical research leadership, and presentations at Duke clinical research community events. Currently, the community is continuously updated on new tools and new versions.

To make it as smooth as possible for managers to incorporate the new tools into their onboarding processes, the WE-R team launched an on-demand training module, a Clinical Research Onboarding Manager Guide webpage, and no-cost onboarding consultations. Information about these is publicly available on the DOCR Onboarding and Training for Clinical Research Professionals website. Managers can request the onboarding consultation with DOCR to discuss their current onboarding process, review the central onboarding tools, and receive guidance on incorporating the program into their CRU’s current onboarding practices. The WE-R team provides continuous support as needed and managers can request as many consultations as they need.

### 4.4 Tracking program use

Because CRUs independently manage hiring and oversight of their CRP employees, we do not require the use of our centralized tools for all new CRP employees. Instead, expectations for use are set by the leadership within each CRU. With the decentralized nature of our workforce, tracking the use of each element requires unique strategies. Completion of the Express Start modules is tracked via the Duke Learning Management System. Onboarding Learning Plan use is more difficult to ascertain. Upon receiving the OLP from their manager, employees first engage with a link to a REDCap survey to manually “Register Use” of the plan. For the Engagement Activity Packets, we track use via the number of downloads of each packet from Duke Box.

## 5 Program assessment

Two years following the initiation of the phased launch, and adaptation as described in section 4.1, we employed a new evaluation strategy. To evaluate satisfaction with the suite of tools, three separate surveys were disseminated to managers and staff who reportedly used the tools between August 2021 and July 2022. An Express Start Employee survey was sent to employees who completed the Express Start modules in the Duke Learning Management System. Managers and staff received a role-specific OLP and Engagement Packet survey if they reported the use of an OLP. Survey totals and response rates have been provided below for all three surveys.

- Express Start Employee Survey: 53% response rate (sent 185/responded 98 – CRC, 44; CRNC, 18; CRS, 21; RC, 8; RPL, 5)
- Onboarding Learning Plan and Engagement Packet Employee Survey: 56% response rate (sent 102/ responded 57 – CRC, 30; CRNC, 15; CRS, 8; RC, 3; RPL, 1)
- Onboarding Learning Plan and Engagement Packet Manager Survey: 71% response rate (sent 52/ responded 37)

**Figure 3:**
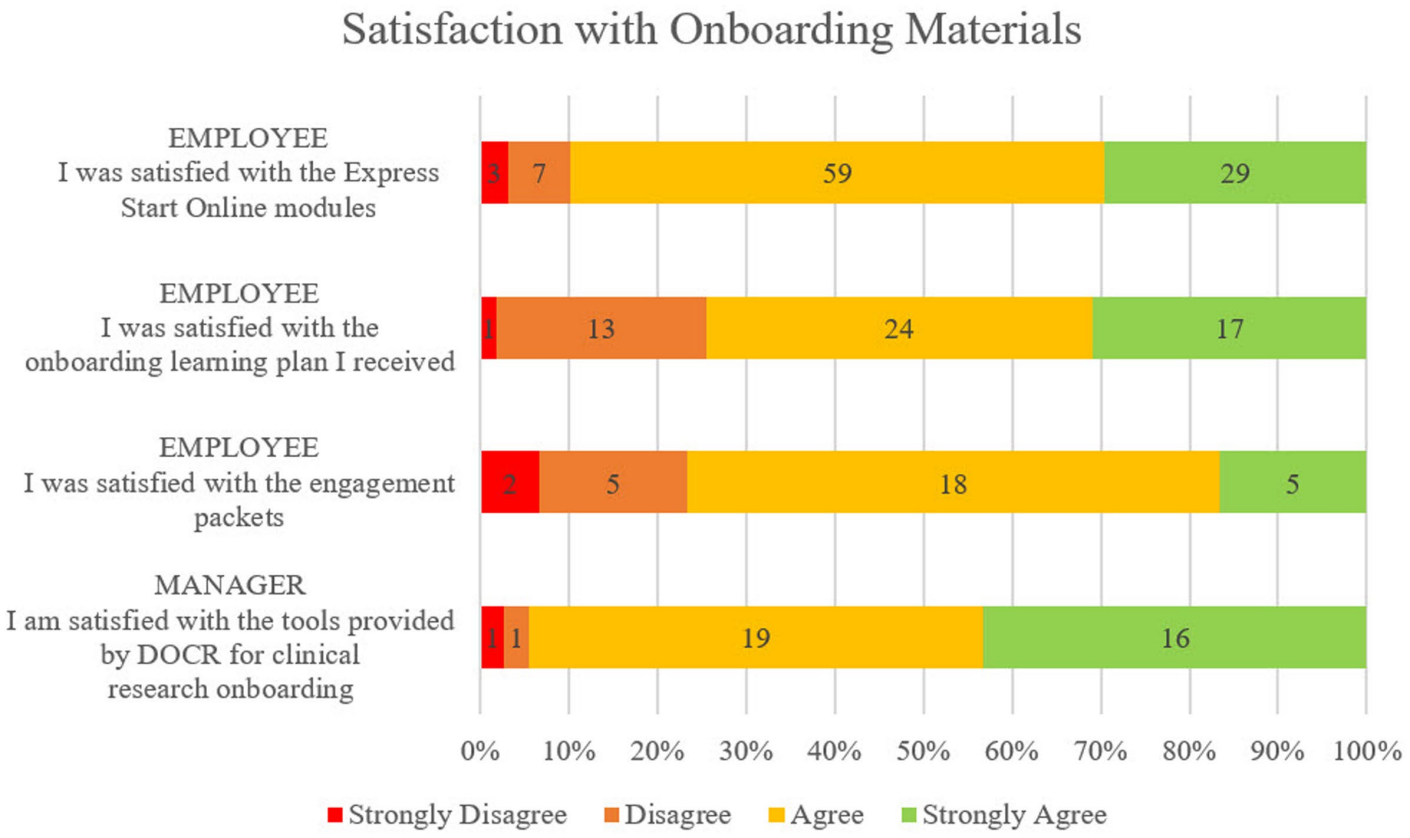
Satisfaction with onboarding components from the employee perspective and the manager perspective on a 4-point scale from Strongly Disagree to Strongly Agree. Employees recorded satisfaction with Express Start, Onboarding Learning Plan, and Engagement Activity Packets. Managers recorded satisfaction with the full suite of tools provided for their use.

Survey respondents represented 21 of the 23 Duke CRUs in operation during the assessment period as well as each of the included CRP jobs, with a majority of respondents in the CRC role that makes up the largest percentage of our workforce. As displayed in Figure 3, 70% or more respondents either agreed or strongly agreed that they were satisfied with the onboarding tools they used. 30 out of the 57 respondents confirmed completion of the Engagement Activity Packet component with 25 of those 30 agreeing or strongly agreeing that they were satisfied with them. All 30 employees agreed or strongly agreed that the engagement activities included were useful for their role and 28 of the 30 employees agreed or strongly agreed that the engagement activities helped them apply what they have learned on the job. When compared, those employees who answered agree or strongly agree to “my manager played an active role in my onboarding activities” or “my manager and I thoroughly reviewed the engagement packets together” more often reported satisfaction with onboarding tools than those who did not feel that their manager was engaged.

In addition to expressing overall satisfaction with the tools, managers overwhelmingly agreed or strongly agreed that the tools were easy to find (92%), clear and easy to use (100%), and saved them time preparing for and onboarding their new employees (95%). Most managers agreed or strongly agreed that the central onboarding tools closely matched the roles they were onboarding (90%).

Manager satisfaction with the tools is evident in their use across CRUs despite the absence of any central requirement to use them. Table 2 captures information about employee completion of each onboarding component during the assessment period. From August 2021 to July 2022, 305 employees were hired, transferred, or reclassified into one of the CRP positions. Of those 305 employees, 190 (62.3%) completed all of the Express Start modules for their role, and 120 (39.3%) registered their use of an OLP. The completion percentages are higher when we consider only hires who are brand new to Duke. Of the 145 total hires new to Duke, 110 (75.9%) completed Express Start, and 64 (44.1%) registered the use of an OLP. These data do not include Research Program Leaders, because an OLP and Express Start were not yet available for the role during this period. The higher uptake of the Express Start modules may stem from the Learning Management System which automatically tracks and records completion. Data about OLP usage, on the other hand, is tracked via voluntary submission of a REDCap registration form by the employee or manager and numbers may underrepresent usage if downloaded and used without registration.

**Table 2:** The count (*N*) of clinical research professionals (CRPs)* who joined the workforce at Duke within the assessment period (August 2021 – July 2022).

Type of Hire	*N*	Completed express Start	Registered onboarding learning plan
Hires new to Duke	145	110 (75.9%)	64 (44.1%)
Transfers and reclassifications within Duke	160	80 (50%)	56 (35%)
All	305	190 (62.3%)	120 (39.3%)

The rightmost columns illustrate the number and percentage of those hires who completed Express Start and/or registered use of an Onboarding Learning Plan for their role.*This table does not include hire data for research program leaders because the onboarding tools for this role were in development during the assessment period.

A few themes emerged from the analysis of 19 employee qualitative comments. Comments were mainly constructive, and themes were consistent regardless of stated satisfaction with the learning plan received. Themes included document issues (e.g., redundancy and broken links) (6), uninvolved/unprepared managers (12), and lack of and/or need for shadowing (6). Comments around the need for shadowing, lack of manager involvement, and not customizing templates indicate mismanagement of the process by managers and failure to use the tools as intended. This emphasizes the importance of additional manager training, which we have prioritized developing since the assessment. The OLP template, by itself, does not provide everything an employee needs to be successfully onboarded. Manager or mentor involvement in tailoring the onboarding experience is an essential element that can only be supplemented by any training tools.

Manager comments were overwhelmingly positive. Among the available comments from 15 managers, themes included general appreciation of the tool (10), customizability (3), helpfulness of engagement activities (3), and comprehensiveness of the tools (2). Constructive comments included the need for a plan that caters to senior-level roles and the lack of time available to them for effective onboarding. A few managers indicated the need for something that was already included in the toolkit (e.g., shadowing ideas and a customizable timeline), further confirming the need for additional manager training on using these tools effectively. Only two managers disagreed that they were satisfied with the tools and neither provided comments, therefore a thematic analysis of dissatisfaction was not possible.

## 6 Reflections, challenges, and future opportunities

### 6.1 Reflections

The program assessment described above illustrates the utility of a centrally offered, standardized onboarding program in meeting the needs of newly hired CRPs. Survey results indicate a high degree of satisfaction with the centrally offered onboarding tools from both new employees and their managers. A majority expressed that the competency-based onboarding tools aligned with their job duties and prepared them to be successful in their work, emphasizing their clarity, ease of use, and time-saving benefits. Managers indicated a high level of satisfaction with the tools, and their positive feedback aligns with their proactive use of the onboarding resources despite the lack of requirement.

The completion rates for the Express Start modules and Onboarding Learning Plans reveal a noteworthy initial adoption among new hires. Despite the voluntary nature of the program and the constraints on their time, both managers and employees actively opt to use these tools. This underscores the motivation of CRPs to undergo comprehensive training, emphasizing their commitment to succeeding in their roles and delivering high-quality work. At the same time, it reflects managers’ need for effective onboarding tools to facilitate the integration of new hires into their teams. Taken together our data indicate that a competency-based centralized onboarding program can be standardized for clinical research job classifications while still accommodating the unique requirements of distinct research areas.

### 6.2 Challenges and opportunities

Because of our federated research structure, we have limited ability within the central WE-R team to control how the program tools are used within each CRU. There is a clear need for ongoing manager training and outreach about the availability of the tools themselves and onboarding best practices. We have implemented onboarding consultations to help managers apply our tools and are expanding manager training opportunities. However, manager time may continue to be a barrier to effective onboarding for some, given general competing priorities for time and effort. Although we are encouraged that the onboarding tools are saving managers’ time and reducing burden, we are cautious that this may reflect inappropriate use of the tools, replacing meaningful manager engagement rather than enhancing and fostering mentoring relationships with new staff. Because of this, we are continuing outreach across CRU leadership and management communities to promote manager engagement during the onboarding process and to identify remaining educational gaps.

Another challenge is the time-intensive maintenance of tools. While relatively low maintenance compared with labor-intensive fully centralized onboarding models, this program requires one full-time employee to develop and at least 50% effort to maintain post-implementation. During the start-up phases, an instructional designer/project manager FTE as well as 10%–20% effort from the steering team was needed. For sites that have fewer resources, we offer the use of our publicly available toolkit without cost so that resources can be minimized to those required for adapting and implementing the educational framework at their site.

Finally, because we strove to produce a low-technology program that could be widely adopted without technology-related costs, our tracking mechanisms for some components of the program are limited. To reflect more directly on whether this onboarding program contributes to employee competency development and career advancement over time will require additional tracking mechanisms and partnerships with CRUs.

### 6.3 Leadership and manager onboarding and training

As a next step for the onboarding program, we will focus on building out tools for CRPs hired into senior-level positions. The study conduct competencies required of these positions are captured in the existing tools; however, there is a need for additional content to be added for team lead and manager roles. Future offerings for managers will cover use of onboarding and training tools for their employees, best practices in hiring and professional advancement, and critical management and leadership skills. Introducing the onboarding tools and best practices during onboarding for senior staff, who will primarily manage and onboard future CRPs, will help improve awareness and alleviate some of the program implementation challenges presented above.

### 6.4 Adding a social component to onboarding: new hire cohorts and mentoring

There are three main components of successful onboarding; organizational, technical, and social (21). Express Start and CRU-specific training address the organizational component by showing employees how Duke functions and where they fit into clinical research. Technical aspects are covered within the Onboarding Learning Plans and competency-based Engagement Packets that help establish fundamental competency and allow employees opportunities for practice. In a new labor era where CRPs work in many different settings, including their homes and community settings, the social component is inherently important. To supplement our existing onboarding tools, we have begun piloting a New Hire Cohort and Mentoring Program that includes 6 months of bi-weekly group meetups with an experienced mentor to facilitate discussions and monthly foundational live training sessions. Our intention with this program is to provide a social element to onboarding, build a collaborative community across clinical research positions and units, and provide a professional growth opportunity for experienced clinical research staff.

### 6.5 Final thoughts

The alignment of the CRP onboarding program at Duke with the Joint Task Force for Clinical Trials Competency (JTFCTC) framework, has provided a structured approach to introduce CRPs to competency-based thinking early in their career (6). This alignment allows for consistent growth within the established framework, promoting continuous competency-based educational opportunities and facilitating competency-based career advancement (8).

At this time, we believe there are no published or disseminated tools from other institutions for a similar on-demand and competency-based onboarding program aligned with the JTFCTC framework for CRPs. However, since launching our Onboarding Toolkit^3^ in August 2023 for other institutions to access and download, 35 different Academic Medical Centers have requested access to the tools described in this paper. This, alongside our collaborative Un-Meeting findings (5), demonstrates that there is a critical need for standardized, competency-based, on-the-job training to develop early talent among newly hired site CRPs. Readily available, easily adaptable, and broadly accessible tools, such as ours bridge a critical gap toward improving study quality and building a stronger CRP workforce.

## Data availability statement

The raw data supporting the conclusions of this article will be made available by the authors, without undue reservation.

## Author contributions

JC: conception and design of work, data acquisition and interpretation, and publication draft and revision. CD: conception and design of work, analysis and interpretation of data, and publication revision. DH: conception and design of work and publication draft. DS: conception of work, interpretation of data, and publication revision. SF: conception and design of work, data acquisition and interpretation, and publication draft and revision. All authors contributed to the article and approved the submitted version.

## Footnotes

^1^ https://medschool.duke.edu/research/research-support/research-support-offices/duke-office-clinical-research-docr/workforce-0#toolkit

^2^ http://box.com/

^3^ https://medschool.duke.edu/research/research-support/research-support-offices/duke-office-clinical-research-docr/workforce-0#toolkit
