# Supplementary material for: Development and implementation of an on-demand competency-based onboarding program for clinical research professionals in academic medicine
Source: Front Med (Lausanne). 2023 Dec 22;10:1249527. doi: 10.3389/fmed.2023.1249527 (PMC10771843; doi:10.3389/fmed.2023.1249527)
Supplement: Supplementary file 1 [file Table_1.DOCX]

Supplementary Material

Development and Implementation of an On-Demand Competency-Based Onboarding Program for Clinical Research Professionals in Academic Medicine

# Supplementary Figures and Tables

# Supplementary Table A. A list of Express Start online modules that are assigned to each clinical research role with associated module descriptions.

| **Role** | **Express Start Module & Description** |
| --- | --- |
| Clinical Research Specialist | **What is a CRS at Duke?**  This course covers general roles and responsibilities of a Clinical Research Specialist (CRS) and Clinical Research Specialist Sr. (CRS Sr.) at Duke. In this course you will learn about the expectations of the organization in your role, career progression opportunities, and the roles of other study team members. |
|  | **Navigating Clinical Research at Duke**  This course provides an overview of the general organizational structure of Duke, central research support offices, and the structure of a Clinical Research Unit (CRU). |
|  | **Introduction to the Clinical Research Workflow and Systems**  This course provides a basic overview of the clinical research workflow at Duke and highlights the specific electronic systems that support clinical research management. It is helpful for the Clinical Research Specialist role to be aware of these workflows and available systems for clinical research. |
| Clinical Research Coordinator | **What is a Clinical Research Coordinator at Duke?**  This course covers general roles and responsibilities of a Clinical Research Coordinator (CRC) or Clinical Research Nurse Coordinator (CRNC) at Duke. In this course you will learn the expectations of the organization in your role, career progression opportunities, and the roles of other study team members. NOTE: CRNCs have an additional module that reviews their clinical/nursing related responsibilities and is included in CRNC Express Start and as a separate stand-alone module. |
|  | **Navigating Clinical Research at Duke**  Same description as course with duplicate-title above. |
|  | **Introduction to the Clinical Research Workflow and Systems**  Same description as course with duplicate-title above. |
|  | **Getting Started with Your Clinical Research Portfolio of Studies**  This course provides tips and tricks for becoming familiar with your clinical research portfolio of studies. We will share tools and resources to help Clinical Research Coordinators and Clinical Research Nurse Coordinators get started participating in the management of your clinical research studies. |
| Clinical Research Nurse Coordinator | **Modules 1 – 4 are the same as CRC above.** |
|  | **CRNC Nursing Responsibilities and Competencies**  This course provides Duke Clinical Research Nurse Coordinators (CRNCs) with an introduction to their unique role, why it is important, and how nursing skills and responsibilities tie into this clinical research role. |
| Regulatory Coordinator | **What is a Regulatory Coordinator at Duke?**  This course covers general roles and responsibilities of a Regulatory Coordinator at Duke. In this course you will learn the expectations of the organization in your role, career progression opportunities, and the roles of other study team members. |
|  | **Navigating Clinical Research at Duke**  Same description as course with duplicate-title above. |
|  | **Introduction to the Clinical Research Workflow and Systems**  This course provides a basic overview of the clinical research workflow at Duke and highlights the specific electronic systems that support the job functions of a regulatory coordinator involved in clinical research management. |
|  | **Institutional Regulatory Policies and Procedures in Clinical Research**  This course provides Duke employees engaged in clinical research with information about regulations, guidelines, and policies for conducting clinical research at Duke University. |
| Research Program Leader | **What is a Research Program Leader at Duke?**  This course covers general clinical research related roles and responsibilities of a Research Program Leader (RPL) at Duke. In this course you will learn about the general organizational expectations of the RPL role and the variation that exists in the RPL role. This course also includes information about career progression opportunities, and the roles of other study team members you may work with frequently. |
|  | **Navigating Clinical Research at Duke**  Same description as course with duplicate-title above. |
|  | **Intro to The Clinical Research Workflow and Systems at Duke**  This course provides a basic overview of the clinical research workflow at Duke and highlights the specific electronic systems that support clinical research management. These systems may be used regularly by the Research Program Leader in some capacity. |
|  | **Getting Started With Your Clinical Research Portfolio**  This module will provide tips and tricks for becoming familiar with your clinical research portfolio of studies. RPLs will receive tools and resources to help you get started participating in the management of your clinical research studies. |
|  | **Project Management Fundamentals**  This module provides RPLs with the fundamental framework of project management within a clinical research context. In this course, you'll review the fundamentals of project management, including how to create a project plan, define project activities, communicate schedules and deadlines effectively, and report on the status of projects. |
| Research Practice Manager | **Navigating Clinical Research at Duke**  This course provides the general organizational structure of Duke, central support offices, and an overview of a Clinical Research Unit (CRU) and Oversight Organization (OO). |
|  | **My Role and My Team’s Role at Duke**  This course introduces new Research Practice Managers (RPMs) to the general role and certain responsibilities related to the role. This course will also introduce you to the structure of your teams and the general roles you can expect to manage within your CRU. The course is intended for Research Practice Managers or Assistant Research Practice Managers (ARPMs) who will be delegated responsibilities by the RPM. |
|  | **Overview of WE-R Initiatives and Tier Advancement**  This course introduces new RPMs and ARPMs to responsibilities related to WE-R initiatives and the tier advancement process as a whole. |
|  | **Introduction to The Clinical Research Workflow at Duke**  This course provides a basic overview of the clinical research workflow at Duke and highlights the specific electronic systems that support clinical research management. |
| CRU Director | **CRU Director Express Start**  This course will acquaint new Clinical Research Unit (CRU) Directors (also referred to as CRU Medical Director) with the structure and expectations of their role within the CRU, the responsibilities of the other roles within the CRU, support available across Duke for clinical research, and other items that are important for the CRU Director to know. |

**
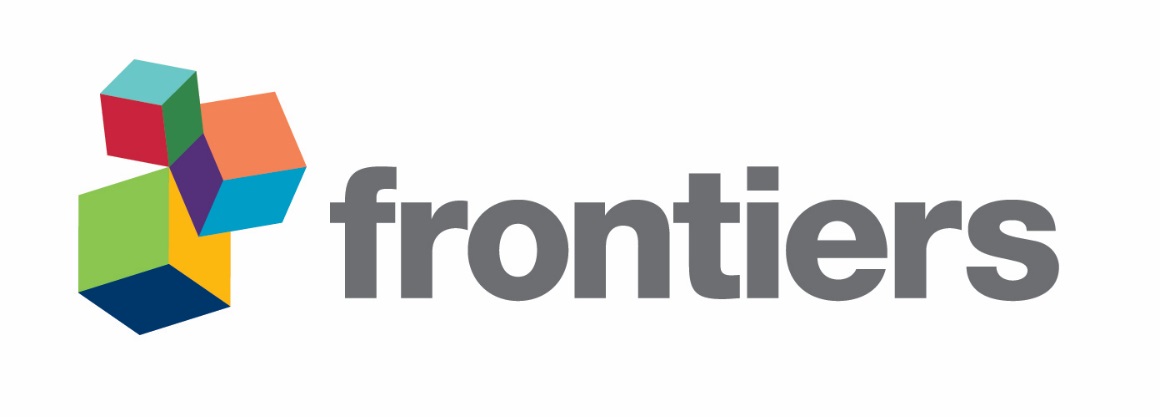
**
